# Multiomic Characterization Reveals a Distinct Molecular Landscape in Young-Onset Pancreatic Cancer

**DOI:** 10.1200/PO.23.00152

**Published:** 2023-11-09

**Authors:** Ifeanyichukwu Ogobuiro, Yasmine Baca, Jennifer R. Ribeiro, Phillip Walker, Gregory C. Wilson, Prateek Gulhati, John L. Marshall, Rachna T. Shroff, David Spetzler, Matthew J. Oberley, Daniel E. Abbott, Hong Jin Kim, David A. Kooby, Shishir K. Maithel, Syed A. Ahmad, Nipun B. Merchant, Joanne Xiu, Peter J. Hosein, Jashodeep Datta

**Affiliations:** ^1^ Department of Surgery, Sylvester Comprehensive Cancer Center, University of Miami Leonard M. Miller School of Medicine, Miami, FL; ^2^ Caris Life Sciences, Phoenix, AZ; ^3^ University of Cincinnati Medical Center, Cincinnati, OH; ^4^ Robert Wood Johnson Medical School, The Cancer Institute of NJ, New Brunswick, NJ; ^5^ Georgetown University, Washington, DC; ^6^ University of Arizona Cancer Center, Tucson, AZ; ^7^ Caris Life Sciences, Irving, TX; ^8^ University of Wisconsin Carbone Cancer Center, Madison, WI; ^9^ The University of North Carolina at Chapel Hill, Chapel Hill, NC; ^10^ Winship Cancer Institute, Emory University, Atlanta, GA; ^11^ Department of Medicine, University of Miami Leonard M. Miller School of Medicine, Miami, FL

## Abstract

**PURPOSE:**

Using a real-world database with matched genomic-transcriptomic molecular data, we sought to characterize the distinct molecular correlates underlying clinical differences between patients with young-onset pancreatic cancer (YOPC; younger than 50 years) and patients with average-onset pancreatic cancer (AOPC; 70 years and older).

**METHODS:**

We analyzed matched whole-transcriptome and DNA sequencing data from 2,430 patient samples (YOPC, n = 292; AOPC, n = 2,138) from the Caris Life Sciences database (Phoenix, AZ). Immune deconvolution was performed using the quanTIseq pipeline. Overall survival (OS) data were obtained from insurance claims (n = 4,928); Kaplan-Meier estimates were calculated for age- and molecularly defined cohorts. Significance was determined as FDR-corrected *P* values (*Q*) < .05.

**RESULTS:**

Patients with YOPC had higher proportions of mismatch repair–deficient/microsatellite instability-high, *BRCA2*-mutant, and *PALB2*-mutant tumors compared with patients with AOPC, but fewer *SMAD4-*, *RNF43-*, *CDKN2A-*, and *SF3B1-*mutant tumors. Notably, patients with YOPC demonstrated significantly lower incidence of *KRAS* mutations compared with patients with AOPC (81.3% *v* 90.9%; *Q* = .004). In the *KRAS* wild-type subset (n = 227), YOPC tumors demonstrated fewer *TP53* mutations and were more likely driven by *NRG1* and *MET* fusions, whereas *BRAF* fusions were exclusively observed in patients with AOPC. Immune deconvolution revealed significant enrichment of natural killer cells, CD8^+^ T cells, monocytes, and M2 macrophages in patients with YOPC relative to patients with AOPC, which corresponded with lower rates of *HLA-DPA1* homozygosity. There was an association with improved OS in patients with YOPC compared with patients with AOPC with *KRAS* wild-type tumors (median, 16.2 [YOPC-*KRAS*^WT^] *v* 10.6 [AOPC-*KRAS*^WT^] months; *P* = .008) but not *KRAS*-mutant tumors (*P* = .084).

**CONCLUSION:**

In this large, real-world multiomic characterization of age-stratified molecular differences in pancreatic ductal adenocarcinoma, YOPC is associated with a distinct molecular landscape that has prognostic and therapeutic implications.

## Teaser

Distinct molecular landscape of #YoungOnset #PancreaticCancer #pancsm using @carisls data from @DrJashDatta #DattaLab @IOgobuiro #CARISPOA & #CPC (in @JCOPO_ASCO)—the parentheses are optional since it will be coming from this handle.

## INTRODUCTION

Pancreatic ductal adenocarcinoma (PDAC) is a highly lethal malignancy with a 5-year survival rate of 12%^1^ and is a leading cause of cancer-related mortality in the United States.^2^ PDAC is typically diagnosed in the seventh decade of life, referred to as average-onset pancreatic cancer (AOPC)^1,3^; however, young-onset pancreatic cancer (YOPC)—defined as diagnosis at age <50 years^4,5^—constitutes 6%-9% of newly detected PDAC and has steadily increased in incidence over the past two decades.^5-12^ Emerging data indicate that smoking,^4,5^ alcohol use,^13^ obesity,^14^ and family history^13,15,16^ are risk factors for YOPC. YOPC also skews toward male sex^7,11^ although rates in women—particularly Black women—are rising faster than in men.^6,7,12^

**CONTEXT**

**Key Objective**
Young-onset pancreatic cancer (YOPC) represents a growing proportion of patients diagnosed with pancreatic ductal adenocarcinoma before age 50 years, with distinct clinical characteristics. Using a large real-world molecular database, we characterized the molecular features underlying these clinical differences between patients with YOPC and patients with average-onset pancreatic cancer (AOPC; 70 years and older).
**Knowledge Generated**
Compared with AOPC, YOPC demonstrated an increased incidence of mutations in DNA repair genes such as *BRCA2* and *PALB2* but lower rates of alterations in oncogenic driver genes, most notably *KRAS.* Among the *KRAS* wild-type cohort, YOPC was more likely driven by *NRG1* and *MET* fusions, but not *BRAF* fusions. YOPC displayed enrichment of distinct immune cell subsets and had lower rates of *HLA-DPA1* homozygosity. Strikingly, patients with YOPC demonstrated improved overall survival that was restricted to the *KRAS* wild-type cohort.
**Relevance**
YOPC is associated with a distinct molecular and immune landscape that could inform targeted therapies for these patients.

The heterogeneity in the molecular landscape of PDAC that underpins its broad range of tumor phenotypes is one of the driving forces for suboptimal outcomes despite modern multimodal therapy.^17^ However, clinically annotated tumor profiling database studies such as the Know Your Tumor study have demonstrated that patients with PDAC experience longer survival when receiving therapies matching actionable mutations compared with nonmatched therapies.^18^ Moreover, The Cancer Genome Atlas analysis of PDAC revealed that, excluding *KRAS* and *CDKN2A*, 42% of patients could be candidates for molecularly informed clinical trials.^19^ The increasing armamentarium of precision medicine approaches for patients with PDAC emphasizes the critical need to understand tumor-level molecular differences between patients with YOPC and AOPC, which might inform personalized therapy in this subset of patients.

Efforts to describe molecular differences between YOPC and AOPC have been hampered by a lack of real-world, large-scale matched genomic and transcriptomic data, leading to conflicting conclusions between studies. For instance, Raffenne et al^3^ found no substantial differences in the mutational landscape between patients with YOPC and AOPC, whereas others have identified higher *SMAD4* mutation rates, increased activation of the TGF-β pathway,^20^ and differential expression of *CDKN2A* and *FOXC2* in YOPC compared with AOPC.^21^ Despite these differences, some unifying signals have emerged, particularly that patients with YOPC harbor fewer oncogenic somatic *KRAS* mutations but more pathogenic germline mutations than patients with AOPC.^16,19,20^ Further complicating our understanding of this question are the conflicting survival outcomes observed in these studies, with many indicating that patients with YOPC have improved survival,^9,11,22,23^ but others showing either shorter or no difference in survival compared with patients with AOPC.^3,5,10,15,20,24^ Together, these results illustrate gaps in our understanding of the genomic and transcriptomic correlates underlying clinical differences between patients with YOPC and AOPC.

In the present study, we analyzed a real-world multi-institutional cohort of 2,430 sequenced tumors—including 292 YOPC—to characterize the distinct molecular landscape associated with YOPC compared with AOPC and better understand molecular correlates underlying the divergent clinical outcomes in patients with YOPC.

## METHODS

### Patient Samples

Two thousand four hundred thirty histologically confirmed PDAC samples were identified in the Caris Life Sciences database (Phoenix, AZ) with matched DNA sequencing, whole-transcriptome sequencing (WTS), and immunohistochemistry (IHC) data. We stratified these specimens into YOPC (younger than 50 years at diagnosis; n = 292) and AOPC (70 years and older; n = 2,138). Among YOPCs, 179 were metastases and 113 were primary tumors; among AOPCs, 1,167 were metastases and 967 were primary tumors.

### Next-Generation Sequencing

Tumor enrichment was achieved using manual microdissection of formalin-fixed, paraffin-embedded (FFPE) sections that were marked for areas with an at least 20% tumor content. Next-generation sequencing (NGS) was performed on genomic DNA using the NextSeq or NovaSeq 6000 platforms (Illumina, Inc, San Diego, CA). For NextSeq-sequenced tumors, a custom-designed SureSelect XT assay was used to enrich 592 whole-gene targets (Agilent Technologies, Santa Clara, CA). For NovaSeq-sequenced tumors, a hybrid pull-down panel of baits designed to enrich for >700 clinically relevant genes at high coverage and read depth was used, along with a separate panel to enrich for an additional >20,000 genes at lower depth. Genetic variants were detected with >99% confidence and were categorized by board-certified molecular geneticists as previously described.^25^ Tumor mutational burden (TMB)-high was defined as ≥10 mutations/Mb.

### IHC

FFPE sections on glass slides were stained for PD-L1 (clone SP142 [Spring Bioscience, Pleasanton, CA]) using automated staining techniques, per the manufacturer’s instructions, and were optimized and validated per Clinical Laboratory Improvement Amendments/College of American Pathologists and International Organization for Standardization requirements. Staining was identified as positive if its intensity on the membrane of the tumor cells was ≥2+ (on a semiquantitative scale of 0-3: 0 no staining, 1+ weak staining, 2+ moderate staining, or 3+ strong staining) and the percentage of positively stained cells was ≥5%.

### Mismatch Repair Deficiency/Microsatellite Instability-High Status

Multiple test platforms were used to determine mismatch repair deficiency (dMMR)/microsatellite instability-high (MSI-H) status of the tumors profiled, including fragment analysis (FA, Promega, Madison, WI), IHC (MLH1, M1 antibody; MSH2, G2191129 antibody; MSH6, 44 antibody; and PMS2, EPR3947 antibody [Ventana Medical Systems, Tucson, AZ]), and NGS. The three platforms generated highly concordant results as previously reported^26^; in the rare cases of discordant results, dMMR/MSI-H status was determined in the order of IHC, FA, and NGS.

### WTS

mRNA was isolated from manually microdissected areas of FFPE sections with a tumor content of at least 10%. Whole-transcriptome sequencing (WTS) was performed using the Illumina NovaSeq platform (Illumina, Inc, San Diego, CA) and the Agilent SureSelect Human All Exon V7 bait panel (Agilent Technologies, Santa Clara, CA); transcripts per million were reported. Gene fusions were detected using the ArcherDX fusion assay (ArcherDX, Boulder, CO) and Illumina MiSeq platform (Illumina MiSeq, San Diego, CA) as previously described.^27^ Immune cell fractions were calculated from transcriptomic data using quanTIseq^28^ and xCell.^29^ Gene set enrichment analysis (GSEA) and Metascape pathway analysis were performed on WTS data.^30,31^ HLA genotyping was performed using arcasHLA, an in silico tool that infers HLA genotypes from RNA sequencing data.^32^ If a single HLA genotype was detected, the specimen was classified as homozygous, which can occur because of parental homozygosity or HLA loss of heterozygosity.

### Statistical Analysis

Clinicodemographic features were compared using the chi-square test, with *P* < .05 considered statistically significant. Comparative analysis of molecular alterations in the cohorts was analyzed using chi-square or Fisher's exact tests. Tumor microenvironment cell fractions were analyzed among cohorts using nonparametric Kruskal-Wallis testing. Because these closely related cohorts are only differentiated by age, *P* values of < .05 were highlighted as relevant trends. For a more stringent analysis of the differences between AOPC and YOPC, *P* values were corrected for multiple hypothesis testing using the Benjamini-Hochberg method to avoid type I error and adjusted *Q <* .05 was considered statistically significant.

### Clinical Outcomes Data

Real-world overall survival (OS) information was obtained from insurance claims data and calculated from the date of tissue collection to last contact. Kaplan-Meier estimates were calculated for YOPC and AOPC in the entire cohort of patients with clinical data in the Caris CODEai clinicogenomic database (n = 4,928) and stratified by *KRAS* mutation status (n = 3,116 patients with *KRAS* data; *KRAS*^WT^, n = 393; *KRAS*^MUT^, n = 2,723); these numbers differ from the molecular analysis since the database is constantly increasing in size. Significance was determined as log-rank *P* < .05.

### Compliance Statement

This study was approved by the Institutional Review Board at the University of Miami and conducted in accordance with guidelines of the Declaration of Helsinki, Belmont report, and US Common rule. Per 45 CFR 46.101(b)(4), this study used retrospective, deidentified clinical data and no patient consent was necessary from the patients.

### Data Availability

Data presented in this study are not publicly available because of data size and patient privacy but are available on reasonable request from the corresponding author.

## RESULTS

### Clinicodemographic Characteristics

At the time of molecular analysis, 2,430 patient samples were annotated with genomic and transcriptomic data. A total of 4,928 patients had available clinical outcomes data in the most recent query of the Caris CODEai clinicogenomic database, from which Kaplan-Meier curves were generated. Of the 2,430 patients with molecular data, 292 patients (12%) had YOPC, with the median age being 46 years (IQR, 41-48). Among 2,138 patients with AOPC (88%), the median age was 75 years (IQR, 72-79). There was a significant preponderance of male patients (65% *v* 52%; *P* < .05) and current smokers (95% *v* 91%; *P* = .023) in patients with YOPC compared with patients with AOPC, respectively (Table 1).

**TABLE 1.**
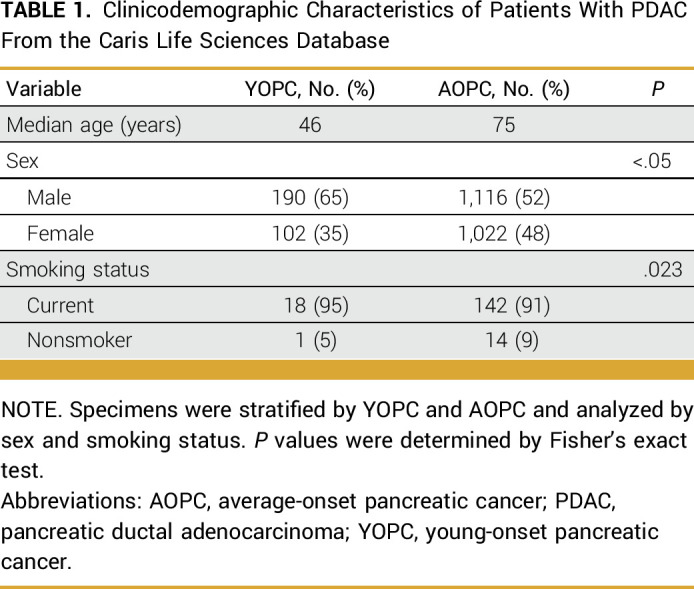
Clinicodemographic Characteristics of Patients With PDAC From the Caris Life Sciences Database

### Comparative Molecular Landscape of YOPC and AOPC

Previous studies have reported differing prevalence of molecular alterations^3,19,20,33,34^ and a preponderance of germline mutations in *BRCA1/2* and MMR genes in patients with YOPC compared with patients with AOPC.^16^ However, direct comparisons between YOPC and AOPC are scarce and have used smaller cohorts.^3,20^ We analyzed clinically relevant pathogenic/likely pathogenic mutations and copy number alterations in tumors from patients with YOPC and AOPC from this real-world cohort (Appendix Table A1).

**FIG 1.**
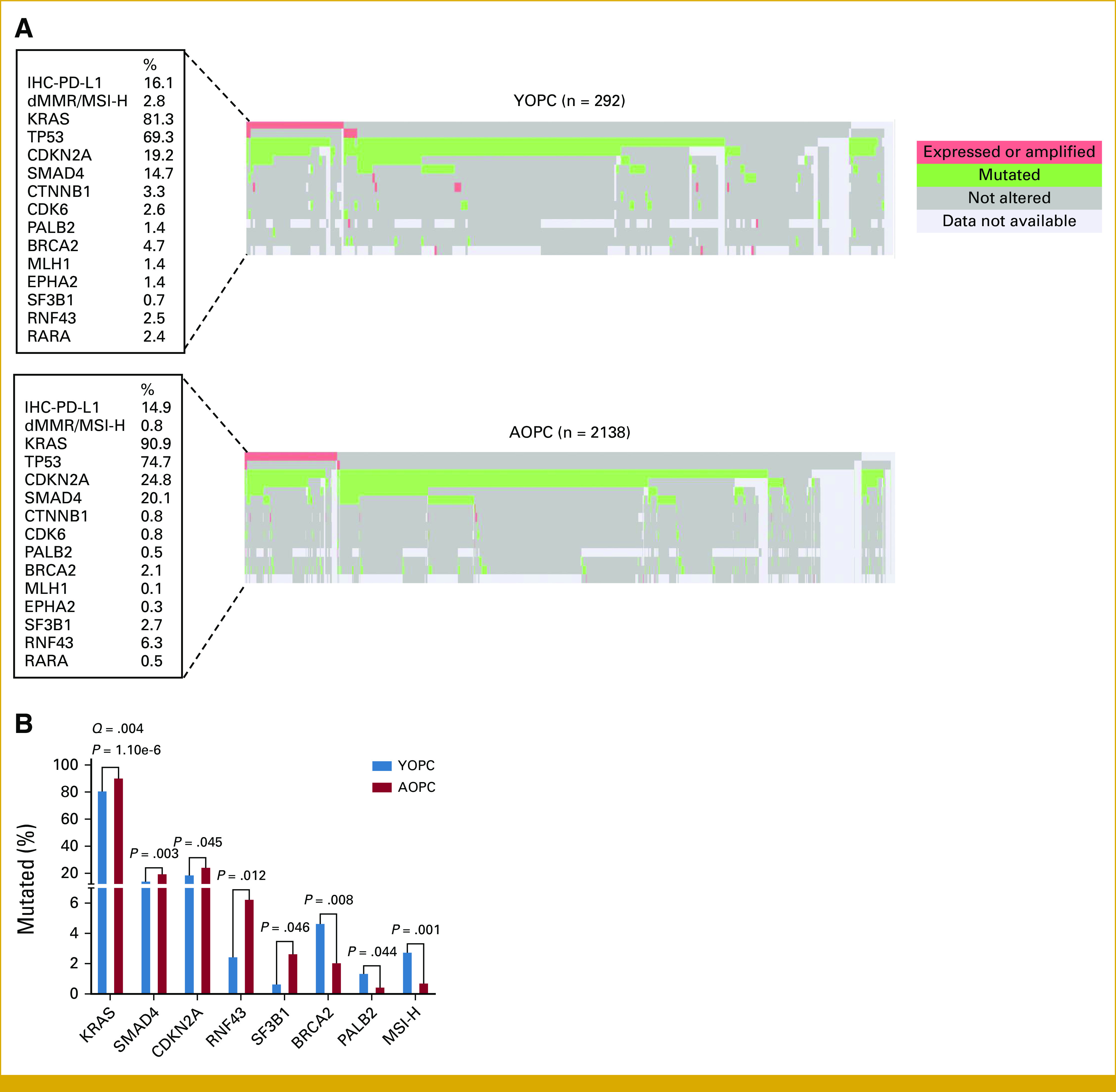
Molecular landscape of YOPC and AOPC. (A) Oncoprints displaying the pathogenic molecular alteration pattern of YOPC (n = 292) and AOPC (n = 2,138). Columns represent tumor samples. Rows represent individual molecular biomarkers, whose percentages in the cohort are described in the boxes to the left of oncoprints. Pink, expressed or amplified; green, mutated; gray; not altered; light gray, data not available. (B) Bar graph showing statistically significant differential molecular alterations in YOPC (blue bars, n = 292) versus AOPC (red bars, n = 2,138). *P* values (chi-square or Fisher’s exact tests) and FDR-adjusted *Q* values are indicated above the compared groups for each molecular alteration. AOPC, average-onset pancreatic cancer; FDR, false discovery rate; YOPC, young-onset pancreatic cancer.

*KRAS* mutations were the most prevalent somatic alterations in both YOPC and AOPC (81.3% and 90.9%), followed by *TP53* (69.3% and 74.7%), *CDKN2A* (19.3% and 24.8%), and *SMAD4* (14.7% and 20.1%; Figs 1A and 1B), respectively. Although germline mutational data were unavailable, patients with YOPC had significantly higher rates of alterations in homologous recombination repair (HRR) genes detected in their tumors, specifically *BRCA2* (4.7% *v* 2.1%; *P* = .008) and *PALB2* (1.4% *v* 0.5%; *P* = .044), compared with patients with AOPC. Patients with YOPC were also noted to have higher rates of dMMR/MSI-H tumors (2.8% *v* 0.8%; *P* = .001) compared with patients with AOPC.

Conversely, patients with AOPC had significantly higher rates of oncogenic *KRAS* mutations compared with patients with YOPC (90.9% *v* 81.3%; *P* = 1.10e-6; *Q* = .004) and significantly higher rates of alterations in *CDKN2A* (24.8% *v* 19.25%; *P* = .045), *SMAD4* (20.1% *v* 14.7%; *P* = .033), *RNF43* (6.3% *v* 2.5%; *P* = .012), and *SF3B1* (2.7% *v* 0.7%; *P* = .046; Figs 1A and 1B).

### Spectrum of Alterations Within *KRAS*^WT^ Tumors in Patients With YOPC and AOPC

**FIG 2.**
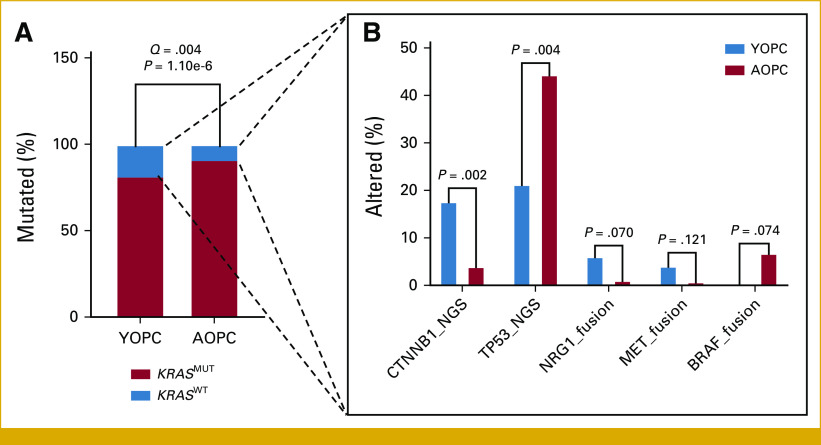
Spectrum of alterations within *KRAS* wild-type YOPC and AOPC tumors. (A) Frequency of *KRAS* wild-type (*KRAS*^WT^; blue, n = 227) and *KRAS*-mutant (*KRAS*^MUT^; red, n = 1,970) tumors in YOPC and AOPC, determined by next-generation sequencing for pathogenic alterations. (B) *KRAS*^WT^ tumors, indicated in blue in (A), were analyzed separately for differences in pathogenic molecular alterations. The spectrum of top mutations and fusions within *KRAS*^WT^ YOPC (blue bars, n = 49-51 [two YOPC-*KRAS*^WT^ patients lacked WTS data for fusions]) and AOPC (red bars, n = 176) is shown, with *P* values (chi-square or Fisher's exact tests) indicated. AOPC, average-onset pancreatic cancer; WTS, whole transcriptome sequencing; YOPC, young-onset pancreatic cancer.

We next dissected the landscape of molecular alterations within the *KRAS* wild-type (*KRAS*^WT^; n = 227 [10.7%]) cohort, given the significant enrichment of *KRAS*^WT^ tumors in patients with YOPC (Fig 2A). Previous studies have implicated the enrichment of mutations in *BRAF*, *CTNNB1*, and alternative RAS pathway genes^19^ in *KRAS*^WT^ PDAC. Accordingly, we observed trends toward increased rates of *CTNNB1* mutations (17.7% *v* 4.0%; *P* = .002) and reduced rates of pathogenic *TP53* mutations (21.3% *v* 44.4%; *P* = .004) in YOPC-*KRAS*^WT^ tumors compared with AOPC-*KRAS*^WT^ tumors. Moreover, YOPC-*KRAS*^WT^ patients demonstrated higher rates of *MET* (4.1% *v* 0.6%; *P* = .12) and *NRG1* (6.1% *v* 1.1%; *P* = .07) fusions compared with AOPC-*KRAS*^WT^ patients, whereas *BRAF* fusions were exclusively concentrated in AOPC-*KRAS*^WT^ compared with YOPC-*KRAS*^WT^ tumors (6.8% *v* 0.0%; *P* = .07; Fig 2B). These results indicate distinct molecular vulnerabilities in *KRAS*^WT^ tumors when stratifying by age of PDAC onset.

### Differentially Regulated Signaling Pathways in Tumor Transcriptomes From Patients With YOPC Versus AOPC

**FIG 3.**
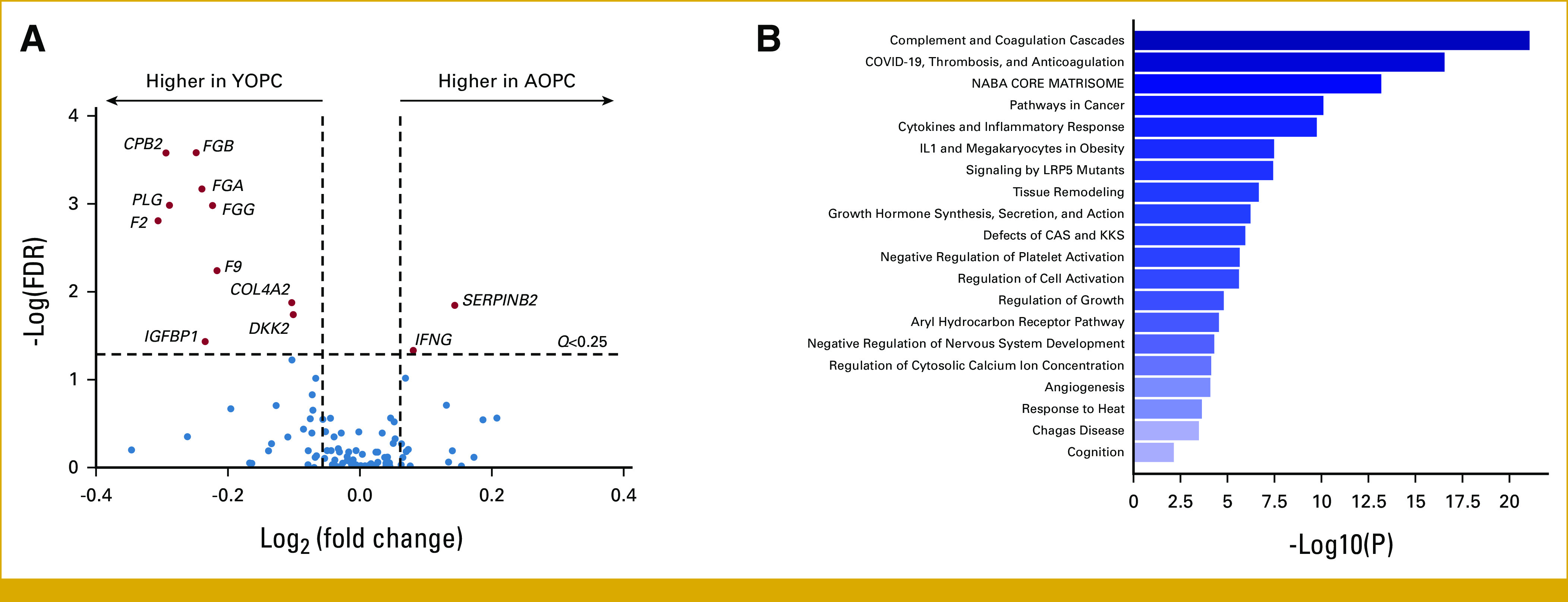
Differentially regulated signaling pathways in YOPC and AOPC. (A) The volcano plot shows DEGs between YOPC (n = 284) and AOPC (n = 2,089), with a cutoff of FDR-adjusted *Q* < .25. Genes to the left indicated in red are significantly higher in YOPC, whereas genes to the right indicated in red are significantly higher in AOPC. (B) Metascape pathway enrichment analysis was performed on 40 DEGs between YOPC and AOPC (*P* < .25). The bar graph indicates canonical signaling pathways and biologic processes differentially enriched in the tumor transcriptomes of YOPC compared with AOPC tumor samples. The *x*-axis indicates statistical significance (–log_10_ *P* value). AOPC, average-onset pancreatic cancer; CAS, contact activation system; DEGs, differentially expressed genes; FDR, false discovery rate; KKS, kallikrein/kinin system; YOPC, young-onset pancreatic cancer.

To better understand how these genomic differences between YOPC and AOPC tumors influence downstream oncogenic and tumor microenvironment signaling, we performed GSEA comparing whole-tumor transcriptomes in YOPC versus AOPC. A relatively narrow number—that is, total of 20—of genes were significantly differentially expressed (*P* < .05; *Q* < .25) between YOPC and AOPC (Fig 3A; Appendix Table A2). The top genes more highly expressed in YOPC included carboxypeptidase B (*CPB2*), plasminogen (*PLG*), prothrombin (*F2*), and genes for fibrinogen alpha and beta chains (*FGA/FGB*), whereas plasminogen activator inhibitor 2 (*SERPINB2*) and interferon gamma (*IFNG*) had significantly higher expression in AOPC. We then used a less stringent *P* value cutoff (*P* < .25) in Metascape pathway analysis to clarify the transcriptomic nuances of these age-stratified PDAC cohorts. This analysis revealed that YOPC tumor transcriptomes were significantly enriched in pathways related to blood clotting cascade, extracellular matrix, cancer pathways, cytokine/inflammatory response, and angiogenesis (Fig 3B).

### Intratumoral Immune Deconvolution and HLA Landscape in YOPC Versus AOPC

**FIG 4.**
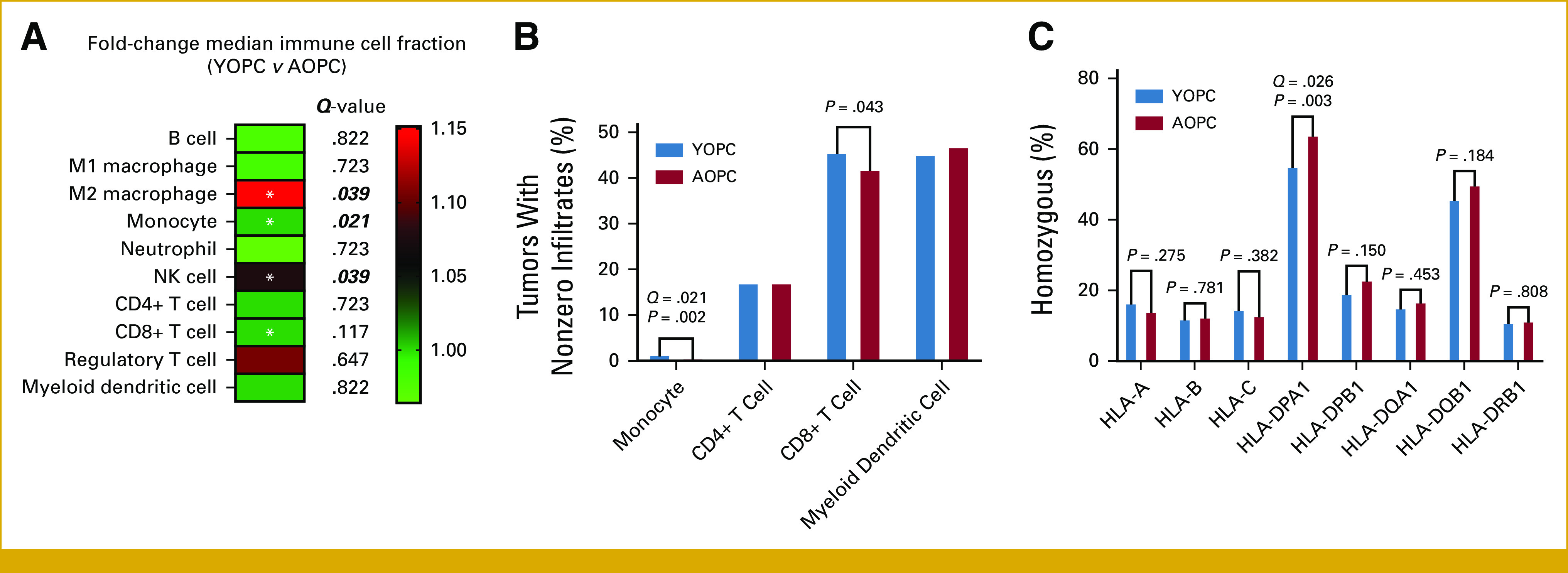
Intratumoral immune populations and HLA landscape in YOPC and AOPC. (A) Computationally inferred intratumoral immune population between YOPC (n = 284) and AOPC (n = 2,089). The heatmap indicates fold change (YOPC *v* AOPC) in median immune fraction according to quanTIseq. *P* values were determined using the nonparametric Kruskal-Wallis test. Asterisks indicate *P* < .05, with *Q* values shown to the right. Italicized/bolded *Q* values indicate *Q* < .05. (B) For cell types with median values of “0” (ie, monocytes, CD4^+^ T cells, CD8^+^ T cells, and myeloid dendritic cells), the percentage of tumors with nonzero immune infiltrates were compared. (C) Differences in HLA landscape inferred from WTS data in YOPC (blue bars, n = 284) compared with AOPC (red bars, n = 2,089). *P* values (chi-square or Fisher's exact test) and FDR-adjusted *Q* values are indicated above compared groups for each HLA gene. AOPC, average-onset pancreatic cancer; FDR, false discovery rate; WTS, whole-transcriptome sequencing; YOPC, young-onset pancreatic cancer.

**FIG A1.**
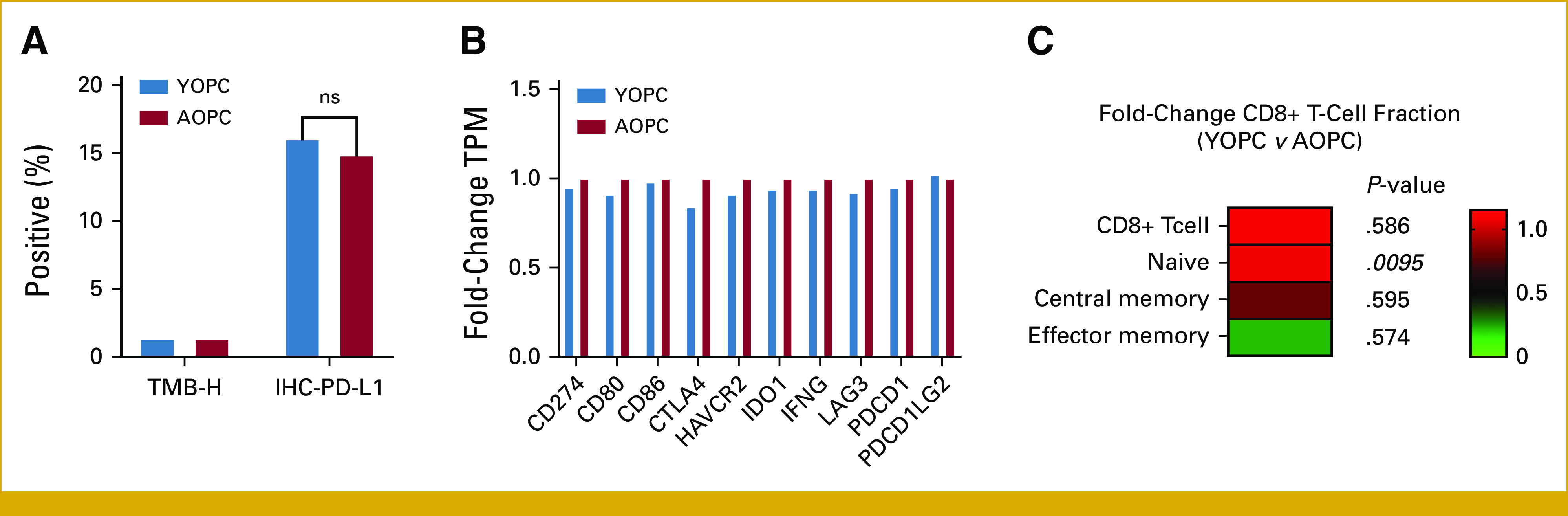
Immune landscape of YOPC and AOPC. (A) Percentage of cases positive for immunotherapy biomarkers TMB-H and PD-L1(+) for YOPC (blue bars, n = 273-292 [a small number of patients lacked PD-L1 IHC or dMMR/MSI-H analysis]) and AOPC (red bars, n = 2,028-2,138 [a small number of patients lacked PD-L1 IHC or dMMR/MSI-H data]). Statistical analysis was performed using the chi-square or Fisher's exact test. (B) Fold-change gene expression levels in TPM of immune checkpoint genes between YOPC (blue bars, n = 284) and AOPC (red bars, n = 2,089). AOPC expression is set to 1 for each gene. There were no statistically significant differences in immune gene expression between YOPC and APOC (determined using the Mann-Whitney U test). (C) Fold-change median CD8^+^ T-cell fractions (YOPC *v* AOPC) calculated by xCell immune deconvolution. *P* value determined using the Kruskal-Wallis test. Significant *P* value (<.05) indicated by italics. AOPC, average-onset pancreatic cancer; dMMR, mismatch repair deficiency; IHC, immunohistochemistry; MSI-H, microsatellite instability-high; TMB-H, tumor mutational burden-high; TPM, transcripts per million; YOPC, young-onset pancreatic cancer.

Because of the enrichment of select pathways and somatic alterations with diverse immunologic repercussions in YOPC, we sought to determine differences in the tumor immune microenvironment between YOPC and AOPC using quanTIseq immune deconvolution.^28^ While there were no significant differences in rates of TMB-high tumors, PD-L1 positivity (via IHC), or immune checkpoint gene expression between the cohorts (Appendix Figs A1A and A1B), there was a statistically significant enrichment in computationally inferred signatures for natural killer (NK) cells (*P* = .009; *Q* = .039), CD8^+^ T cells (*P* = .043; *Q* = .117), M2 macrophages (*P* = .011; *Q* = .039), and monocytic cells (*P* = .002; *Q* = .021) in tumors from patients with YOPC compared with AOPC (Figs 4A and 4B). We then used xCell deconvolution^29^ to further compare CD8^+^ T-cell subsets between cohorts, which revealed no differences in effector or central memory CD8^+^ T cells but demonstrated enrichment of naïve CD8^+^ T cells in tumors from patients with YOPC (Appendix Fig A1C).

To further understand potential major histocompatibility complex determinants that might underlie these immunologic differences between cohorts, we examined HLA-type and locus-specific expression inferred from RNA sequencing data. We observed a significantly decreased rate of homozygosity in *HLA-DPA1* in tumors from patients with YOPC compared with AOPC (55.2% *v* 64.1%; *P* = .003; *Q* = .026; Fig 4C). Taken together, these associative data illustrate potential differences in immunogenicity related to cell-autonomous and/or nonautonomous mediators in tumors from patients with YOPC that might be contributory to differences in clinicopathologic outcomes between patients with YOPC and AOPC.

### OS of Patients With YOPC and AOPC Stratified by *KRAS*^MUT^ and *KRAS*^WT^

**FIG 5.**
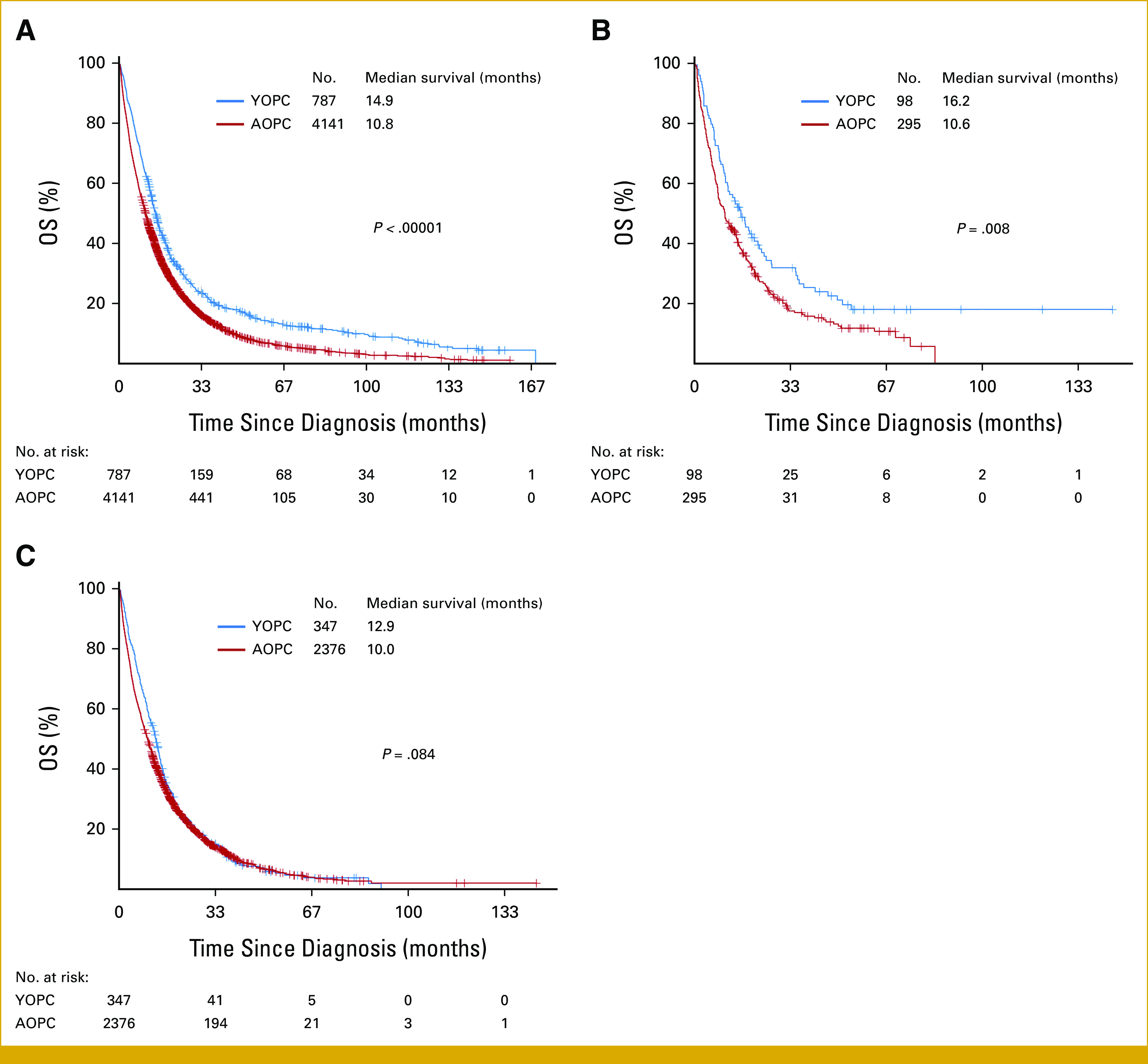
OS of patients with YOPC and AOPC stratified by *KRAS*^MUT^ and *KRAS*^WT^. (A) Kaplan-Meier curves depict the OS of patients with YOPC (blue line, n = 787) versus patients with AOPC (red line, n = 2,753) in the entire PDAC cohort with clinical outcome data (n = 4,141 total). (B) and (C) All PDAC cases with *KRAS* mutation data available were stratified by *KRAS* status. Kaplan-Meier curves depict the OS of patients with YOPC (blue line, n = 98) versus patients with AOPC (red line, n = 295) with *KRAS*^WT^ tumors (n = 393 total; B) and patients with YOPC (blue line, n = 347) and AOPC (red line, n = 2,376) *KRAS*^MUT^ tumors (n = 2,723 total; C). AOPC, average-onset pancreatic cancer; OS, overall survival; PDAC, pancreatic ductal adenocarcinoma; YOPC, young-onset pancreatic cancer.

The Caris CODEai data set included 4,928 patients with insurance claims–related follow-up information, but limited clinicopathologic data precluded stage- and treatment-stratified comparisons between YOPC and AOPC cohorts. Notwithstanding, we observed significantly longer OS in patients with YOPC compared with patients with AOPC (14.9 *v* 10.8 months; *P* < .00001; Fig 5A). Given the differences in frequency of *KRAS*-altered tumors between cohorts, we further analyzed the effect of *KRAS* alteration status on OS. YOPC-*KRAS*^WT^ patients had significantly prolonged OS compared with AOPC-*KRAS*^WT^ patients (16.2 *v* 10.6 months; *P* = .008; Fig 5B). However, there was no difference in OS between patients with YOPC and AOPC with *KRAS*^MUT^ tumors (12.9 *v* 10.0 months; *P* = .084; Fig 5C). These data suggest that survival differences between patients with YOPC and AOPC in the overall cohort may be driven by survival variation specifically in patients harboring *KRAS*^WT^ tumors.

## DISCUSSION

To our knowledge, the present study represents the largest pragmatic molecular comparison of YOPC versus AOPC. Our data reinforce previously observed epidemiologic distinctions between patients with YOPC and AOPC,^4,5,7,11^ specifically its male preponderance and association with active smoking behaviors in patients with YOPC, and conclusively reveal a higher incidence of *KRAS*^WT^ tumors in YOPC. Within this *KRAS*^WT^ subset, we uncovered distinct molecular vulnerabilities when stratifying by age—that is, *MET* and *NRG1* fusions in YOPC-*KRAS*^WT^ and *BRAF* fusions in AOPC-*KRAS*^WT^. Among the unstratified cohort, tumors from patients with YOPC demonstrated higher rates of alterations in HRR genes, higher prevalence of dMMR/MSI-H, and enrichment of NK cells and naïve CD8^+^ T cells. Finally, our data reconcile conflicting previous evidence by demonstrating improved survival in patients with YOPC compared with patients with AOPC, which may not only reflect the reduced prevalence of the virulent oncogenic drivers *KRAS*, *SMAD4*, and *CDKN2A* in tumor genomes of YOPC patients but also be driven by the significantly longer survival of YOPC-*KRAS*^WT^ versus AOPC-*KRAS*^WT^ patients.

While the success of targeted and immune-based therapies has significantly lagged in PDAC compared with other solid tumors, the Know Your Tumor study illustrated the oncologic importance of molecularly matched targeted therapies in patients with advanced PDAC.^18^ To that end, our data provide a biologic map of the distinct molecular vulnerabilities in patients with YOPC that might be exploited therapeutically. While *KRAS* mutations—with their rapidly evolving therapeutic landscape—^35,36^are ubiquitous in the broader cohort, our data reveal novel age-restricted molecular alterations in *KRAS*^WT^ tumors that may be clinically actionable; *NRG1*, *MET*, and *BRAF* fusions each have associated targeted therapies (eg, afatinib, capmatinib, and encorafenib/vemurafenib, respectively).^37-39^ Moreover, given recent data indicating the benefit of polyADP-ribose polymerase inhibitors (PARPi) in patients with germline or somatic mutations in HRR genes,^40^ the enrichment of *BRCA2* and *PALB2* alterations in YOPC tumors suggests that a higher proportion of patients with YOPC may ultimately be eligible for PARPi. Taken together, these data call for heightened awareness among clinician and nonclinician stakeholders of the distinct genomic landscape in patients with YOPC and underscore the importance of routine NGS testing in younger patients presenting with newly diagnosed advanced PDAC to inform potential molecularly targeted therapeutic approaches.

Exploration of the transcriptomes differentially expressed between YOPC and AOPC revealed enrichment of pathways associated with thrombotic cascades, extracellular matrix, cancer pathways, and cytokine/inflammatory response, which suggests possible restriction of tumor immunity in YOPC.^41-43^ Conversely, the significant reduction in *HLA-DPA1* homozygosity—which has been previously associated with dampened antigen presentation and checkpoint blockade efficacy^44^—and associated increases in computationally inferred adaptive immune subpopulations (ie, NK and CD8^+^ T cells) in YOPC suggest a less immunosuppressive and more immunostimulatory microenvironment. While the impact of greater numbers of intratumoral naïve CD8^+^ T cells in YOPC is unclear, higher circulating levels of these cells have been associated with improved prognosis in other solid tumors, for example, non–small-cell lung cancer.^45^ These findings underscore the need for deeper investigation and functional characterization of cell-autonomous and nonautonomous immunologic repercussions in YOPC tumors. The differentially expressed transcriptome we observed in patients with YOPC in the current study, however, is not strongly consistent with previous—albeit underpowered—studies that revealed enrichment in pathways predominantly related to hedgehog signaling and hypoxia in YOPC.^3,20^ This lack of concordance might be attributable to our substantially larger cohort size and/or the inherent heterogeneity of patients enrolled in this pragmatic real-world study capturing data with wide geographic and clinicodemographic variability, which present novel insights into the genotype-immunophenotype chasm in YOPC.^46^

Our study has several limitations. First, while the classification of YOPC and AOPC into age cutoffs of <50 and ≥70-year was informed in previous studies,^3-5^ this arbitrary distinction may underestimate subtle molecular differences in patients with YOPC. Second, while several of the reported genomic differences did not achieve significance by multiple hypothesis testing, we felt it important to report these novel signals with the recognition that our study compares molecular determinants in two closely related PDAC patient populations differentiated solely by a 20-year age gap. Further validation of the subtle molecular features distinguishing these cohorts is warranted. Third, the lack of clinical annotation (eg, performance status, resection status, stage, BMI, and multimodality treatment information) in the Caris CODEai data set precluded our ability to perform multivariable analyses to account for confounding by these clinical parameters.

Given the rise in YOPC diagnosis in recent years,^5-11^ these data are a timely addition to an expanding compendium of molecular taxonomy that highlights the clinical and phenotypic heterogeneities observed in this distinct cohort of patients.^4,5,7,11,16,19-21^ Furthermore, novel genomic and transcriptomic signals observed in tumors from patients with YOPC may offer a putative molecular basis for the divergent clinical outcomes observed in this population. Moving forward, these data could be incorporated into future trial design to allow more precise selection and stratification of patients with YOPC and AOPC for elements of multimodality and/or novel therapies, with the goal of improving contemporary survival outcomes in this lethal malignancy.

## Figures and Tables

**TABLE A1.**
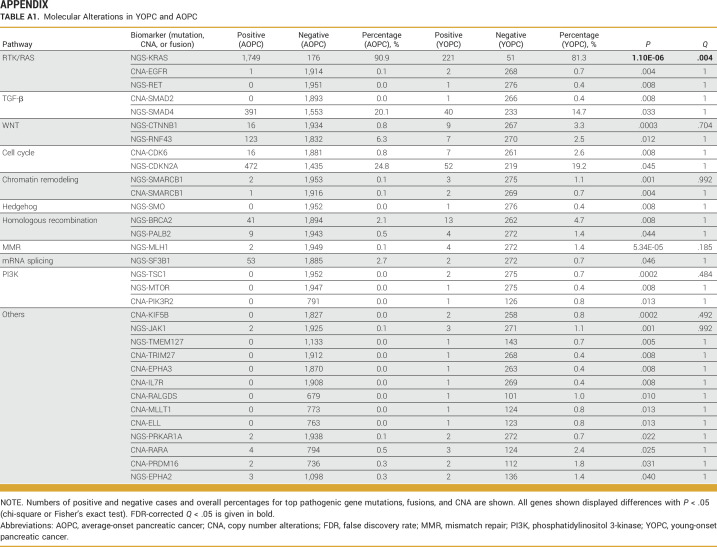
Molecular Alterations in YOPC and AOPC

**TABLE A2.**
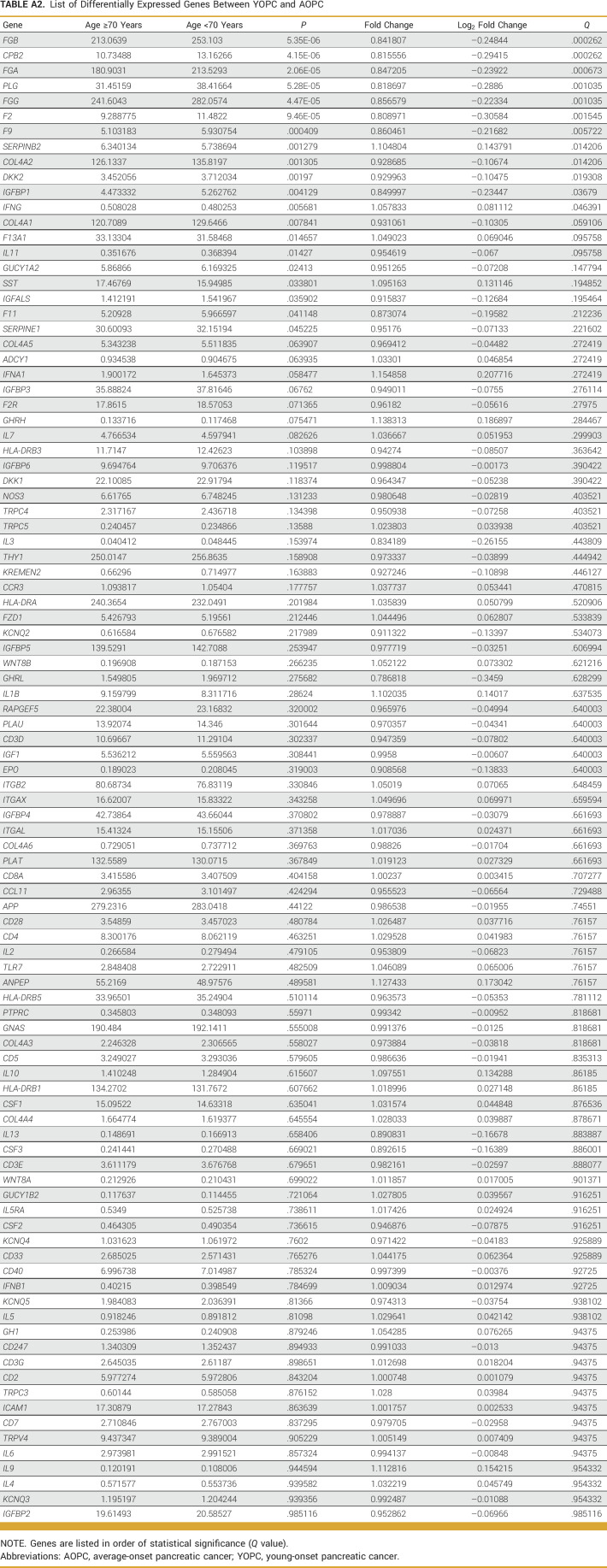
List of Differentially Expressed Genes Between YOPC and AOPC

## PREPRINT VERSION

Preprint version available on medRxiv (https://www.medrxiv.org/node/634311.full).
